# Sex differences in treatment and outcomes of patients with in‐hospital ST‐elevation myocardial infarction

**DOI:** 10.1002/clc.23797

**Published:** 2022-03-07

**Authors:** Julia Stehli, Diem Dinh, Misha Dagan, Ron Dick, Stephanie Oxley, Angela Brennan, Jeffrey Lefkovits, Stephen J. Duffy, Sarah Zaman

**Affiliations:** ^1^ Nursing and Health Sciences, Faculty of Medicine Monash University Melbourne Victoria Australia; ^2^ Epworth HealthCare Richmond Victoria Australia; ^3^ School of Public Health and Preventive Medicine, Centre of Cardiovascular Research and Education in Therapeutics Monash University Melbourne Victoria Australia; ^4^ Department of General Medicine The Alfred Hospital Melbourne Victoria Australia; ^5^ Department of Cardiology Royal Melbourne Hospital Melbourne Victoria Australia; ^6^ Department of Cardiology The Alfred Hospital Melbourne Victoria Australia; ^7^ School of Clinical Sciences at Monash Health Monash University Melbourne Victoria Australia; ^8^ Westmead Applied Research Centre University of Sydney Sydney New South Wales Australia; ^9^ Department of Cardiology Westmead Hospital Sydney New South Wales Australia

**Keywords:** female, in‐hospital STEMI, sex discrepancies, symptom‐to‐device time, women

## Abstract

**Background and Hypothesis:**

Two cohorts face high mortality after ST‐elevation myocardial infarction (STEMI): females and patients with in‐hospital STEMI. The aim of this study was to evaluate sex differences in ischemic times and outcomes of in‐hospital STEMI patients.

**Methods:**

Consecutive STEMI patients treated with percutaneous coronary intervention (PCI) were prospectively recruited from 30 hospitals into the Victorian Cardiac Outcomes Registry (2013−2018). Sex discrepancies within in‐hospital STEMIs were compared with out‐of‐hospital STEMIs. The primary endpoint was 12‐month all‐cause mortality. Secondary endpoints included symptom‐to‐device (STD) time and 30‐day major adverse cardiovascular events (MACE). To investigate the relationship between sex and 12‐month mortality for in‐hospital versus out‐of‐hospital STEMIs, an interaction analysis was included in the multivariable models.

**Results:**

A total of 7493 STEMI patients underwent PCI of which 494 (6.6%) occurred in‐hospital. In‐hospital versus out‐of‐hospital STEMIs comprised 31.9% and 19.9% females, respectively. Female in‐hospital STEMIs were older (69.5 vs. 65.9 years, *p* = .003) with longer adjusted geometric mean STD times (104.6 vs. 94.3 min, *p* < .001) than men. Female versus male in‐hospital STEMIs had no difference in 12‐month mortality (27.1% vs. 20.3%, *p* = .92) and MACE (22.8% vs. 19.3%, *p* = .87). Female sex was not independently associated with 12‐month mortality for in‐hospital STEMIs which was consistent across the STEMI cohort (OR: 1.26, 95% CI: 0.94–1.70, *p* = .13).

**Conclusions:**

In‐hospital STEMIs are more frequent in females relative to out‐of‐hospital STEMIs. Despite already being under medical care, females with in‐hospital STEMIs experienced a 10‐min mean excess in STD time compared with males, after adjustment for confounders. Adjusted 12‐month mortality and MACE were similar to males.

## 1 INTRODUCTION

Coronary artery disease is the leading cause of death worldwide.^1^ Despite advancements in medical therapy and device technology, patients with ST‐elevation myocardial infarction (STEMI) continue to suffer from high mortality.^2^ Rapid reperfusion is key in the treatment of STEMI with time from symptom onset to revascularization closely linked to outcomes.^3^, ^4^, ^5^, ^6^ Two groups of patients with STEMI who demonstrate a particularly poor prognosis are females^7^, ^8^, ^9^, ^10^, ^11^, ^12^ and patients with so‐called “in‐hospital STEMI,” that is, STEMI that happens while the patient is already admitted to the hospital.^13^, ^14^, ^15^ With regard to females, factors associated with poorer outcomes include significantly longer ischemic times in females compared with males^16^, ^17^, ^18^ as well as more bleeding ^19^ and less guideline‐directed medical therapy.^20^, ^21^ Similarly, patients with in‐hospital STEMI have been found to have longer ischemic times compared with patients presenting with out‐of‐hospital STEMI.^13^, ^22^ Further, both female patients, and those with in‐hospital STEMI, are older and have more comorbidities. They are both less likely to undergo invasive diagnostic management as well as to be treated with percutaneous coronary intervention (PCI).^15^, ^20^, ^23^, ^24^

Despite these concerning data, patients with in‐hospital STEMI have been under‐researched, with less than a handful of studies conducted. The topic is of critical importance during the current coronavirus‐19 pandemic: Acute cardiac injury is present in 17% of patients with COVID‐19^25^ and is predictive of in‐hospital mortality in this patient group.^25^ Importantly, STEMIs do occur both as the first sign of COVID‐19 or in‐hospital patients admitted for COVID‐19.^26^

In particular, no research has been undertaken with regard to female patients with in‐hospital STEMI and possible sex disparities in this patient group and as compared with overall STEMI cohorts. The aim of this study was to evaluate sex differences in ischemic times and outcomes of patients with in‐hospital STEMI in a large, multicenter, prospective registry.

## 2 METHODS

From 2013 to 2018, consecutive patients presenting with STEMI who all underwent PCI were prospectively recruited into the Victorian Cardiac Outcomes Registry (VCOR). VCOR is an Australian, state‐based clinical quality registry designed to monitor the performance and outcome of PCI in Victoria. VCOR was established in 2012 and is engaged with all 30 Victorian PCI‐capable hospitals (13 public [i.e., government‐funded] and 17 private) with all patients undergoing PCI or attempted PCI entered into the registry.^27^ VCOR collects baseline demographic, procedural characteristics, in‐hospital, and 30‐day outcome data on all patients who undergo PCI at a given facility through a secure web‐based data collection system. All data entry personnel are registered with VCOR and data are entered in real time as the patient progresses through the hospital admission.^28^ Data integrity is ensured with regular audit activities conducted by the central registry. VCOR is funded by the Victorian Department of Health and Human Services. Twelve‐month mortality was obtained through linkage of the database to the Australian Registry of Births, Deaths and Marriages. The study was approved by the Institutional Human Research Ethics Committee with an opt‐out consent. The investigations were in accordance with the Declaration of Helsinki.

**Figure 1:**
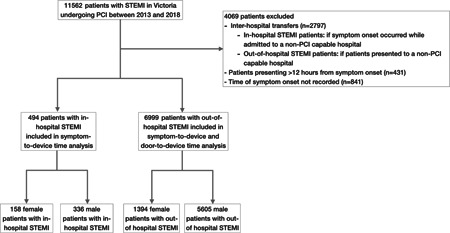
Flowchart displaying inclusion and exclusion of female and male in‐hospital STEMI patients into the time analysis. A total of 7493 patients underwent PCI for the treatment of STEMI of which 494 (6.6%) were in‐hospital STEMI. Of these, 158 patients (31.9%) were female compared with 1394 female patients (19.9%) in the out‐of‐hospital STEMI group. PCI, percutaneous coronary intervention; STEMI, ST‐elevation myocardial infarction

Consecutive patients who received successful or attempted PCI for a STEMI were included. At the time of entering the patient into the VCOR database, STEMI was defined as elevated cardiac biomarkers and new ST‐segment elevation in two or more contiguous leads. The clinical definition was based on a “maximal concentration of Troponin T or I above the MI diagnostic limit on at least one occasion within 24 hours from the index clinical event and ST‐segment elevation in the ECG, equal to the Fourth Universal Definition of Myocardial Infarction.”^29^ In‐hospital STEMI was defined based on the timing of onset of symptoms occurring after the time of hospital admission. As per VCOR definition, the time recorded for symptom onset was when the patients advised the hospital staff of their symptoms. Out‐of‐hospital STEMI included all other STEMI where symptom onset occurred before presentation to hospital. For the analysis of symptom‐to‐device (STD) time, the following patients were excluded from the analysis: (i) patients without a recorded time of symptom onset, (ii) patients who presented for PCI more than 12 h after symptom onset, (iii) patients who had symptom onset while admitted in a non‐PCI capable hospital (for the in‐hospital STEMI), and (iv) patients who presented to a non‐PCI capable hospital (for the out‐of‐hospital STEMI, outlined in Figure 1).

Sex discrepancies in in‐hospital STEMI patients were analyzed and compared with sex discrepancies in out‐of‐hospital STEMI patients for the primary endpoint of 12‐month all‐cause mortality. Secondary endpoints included STD time, 30‐day all‐cause mortality, major adverse cardiovascular events (MACE, consisting of all‐cause death, new or recurrent MI, stent thrombosis or target vessel revascularization), major adverse cardiovascular and cerebrovascular events (MACCE, consisting of MACE and stroke), major bleeding (consisting of Types 3 and 5 according to the Bleeding Academic Research Consortium definition), recurrent MI, new heart failure (defined according to clinical signs), new renal impairment at 30 days, and length of hospital stay.

STD time was defined as the time span between the onset of symptoms and the first device used to re‐establish coronary blood flow during PCI.

The European Society of Cardiology (ESC) guidelines recommend a time of ≤60 min from STEMI diagnosis to device passage in patients presenting to a PCI‐capable hospital.^4^ Additionally, FMC‐to‐electrocardiogram (ECG) acquisition time is recommended at ≤10 min.^4^ A FMC‐to‐ECG acquisition time of ≤10 min and a time span from STEMI diagnosis to reperfusion of ≤60 min would result in an ideal STD time of ≤70 min for in‐hospital STEMIs.^4^ Accordingly, to assess for reperfusion delays, the percentage of female and male in‐hospital STEMI patients achieving an STD time ≤70 min was analyzed. The American Heart Association (AHA) guidelines recommend a first medical contact (FMC)‐to‐device time of ≤90 min in patients presenting to a PCI‐capable hospital.^6^ With no in‐hospital STEMI guidelines available, the time of symptom onset (as patients are already admitted under medical care) can take the place of FMC. Accordingly, to assess for sex discrepancies in reperfusion delays, the percentage of male and female in‐hospital STEMI patients achieving an STD time ≤90 min was analyzed.^6^

Preprocedural creatinine was collected up to 60 days before the PCI and the Cockcroft−Gault formula used to determine the estimated glomerular filtration rate (eGFR). New renal impairment was defined as an absolute rise of serum creatinine ≥44.2 μmol/L or an increase of ≥25% up to 5 days after the index PCI when compared with baseline creatinine. Left ventricular ejection fraction (LVEF) was collected during the index STEMI admission or up to 30‐day post discharge. Moderate LV dysfunction was defined as LVEF < 45%, severe LV dysfunction as LVEF < 30%. New heart failure was defined as clinical evidence of heart failure (i.e., new paroxysmal nocturnal dyspnea, new dyspnea on exertion, chest X‐ray showing pulmonary congestion or dyspnea treated with medical therapy for heart failure). Complex lesions included type B2 and C lesions according to American College of Cardiology/AHA classification guidelines. Mechanical ventricular support included intra‐aortic balloon pump, left ventricular assist device, and/or extracorporeal membrane oxygenation.

Associations in categorical variables were analyzed with *χ* ^2^ or Fisher's exact tests as appropriate and expressed as number and percentage. Continuous variables were analyzed with a *t *test and expressed as mean and standard deviation. Since the distributions of presentation and revascularization times were highly skewed, their data were log‐transformed for analysis and back to determine an estimated geometric mean. Along with sex, variables determined a priori to be included in the multivariate models were age, diabetes, eGFR, previous PCI and/or coronary artery bypass grafting, history of cerebrovascular disease, LVEF, out‐of‐hospital and in‐hospital cardiac arrest, cardiogenic shock, graft lesions, stent thromboses, and occurrence time of symptom onset. The variables forced into and retained in the model were determined based on prior literature and experience that these factors are known to influence MACE and all‐cause mortality.

To investigate the relationship between sex and 12‐month mortality outcomes for out‐of‐hospital STEMI patients compared with in‐hospital STEMI patients, interaction was utilized.

A *p* value <.05 was considered statistically significant for all analyses. Statistical analyses were performed using Stata version 14.

## 3 RESULTS

A total of 7493 patients underwent PCI for the treatment of STEMI of which 494 (6.6%) were in‐hospital STEMI. Of these, 158 patients (31.9%) were female compared with 1394 female patients (19.9%) in the out‐of‐hospital STEMI group (outlined in Figure 1). Table 1 shows the baseline demographic and clinical characteristics. Females with in‐hospital STEMI were significantly older (69.5 vs. 65.9 years, *p* = .003) than males but there were no differences in comorbidities and treatment with anticoagulants.

**Table 1:** Baseline characteristics according to sex and in‐hospital versus out‐of‐hospital STEMI

	In‐hospital STEMI (*n *= 494)			Out‐of‐hospital STEMI (*n* = 6999)		
	Females, *n* = 158 (31.9%)	Males, *n* = 336 (68.0%)	*p *value	Females, *n* = 1394 (19.9%)	Males, *n* = 5605 (80.0%)	*p *value
Age (years)	69.5 (13.5)	65.9 (12.1)	.003	67.6 (13.3)	61.1 (12.1)	<.001
BMI (m^2^/kg)	28.2 (24.1, 32.7)	27.5 (24.7, 30.7)	.15	27.1 (23.5, 31.2)	27.7 (24.8, 30.4)	.002
Diabetes, *n* (%)	37 (23.4)	74 (22.0)	.73	252 (18.1)	838 (15.0)	.004
eGFR (ml/min) < 45 ml/min, *n* (%)	30 (19.87)	48 (14.77)	.162	238 (20.9)	262 (5.7)	<.001
Moderate‐severe LVEF impairment, *n* (%)	45 (31.2)	83 (28.0)	.487	346 (26.7)	1555 (29.5)	.046
Previous CABG and/or PCI, *n* (%)	55 (34.8)	162 (48.2)	.005	140 (10.0)	771 (13.7)	<.001
Cerebrovascular disease, *n* (%)	12 (7.6)	23 (6.8)	.76	63 (4.5)	158 (2.8)	.001
Peripheral vascular disease, *n* (%)	8 (5.1)	28 (8.3)	.19	28 (2.0)	101 (1.8)	.61
Oral anticoagulant therapy, *n* (%)	11 (7.0)	20 (6.0)	.67	51 (3.7)	129 (2.3)	.004
Onset of symptoms 7 a.m.−8 p.m., *n* (%)	94 (62.7)	204 (62.8)	.98	872 (62.6)	3662 (65.3)	.052
Pre‐procedure cardiogenic shock, arrest or intubation, *n* (%)	30 (19.0)	47 (14.0)	.15	156 (11.2)	639 (11.4)	.83
Pre‐hospital ECG notification, *n* (%)	—	—		788 (56.6%)	3205 (57.2%)	.68

Abbreviations: BMI, body mass index; CABG, coronary artery bypass grafting; ECG, electrocardiogram; eGFR, estimated glomerular filtration rate; LVEF, Left ventricular ejection fraction; PCI, percutaneous coronary intervention; STEMI, ST‐elevation myocardial infarction.

Female and male in‐hospital STEMI patients had similar rates of stent thrombosis (17.1% vs. 16.4%). Female in‐hospital STEMI patients were significantly less likely to receive statins (*p* = .030) and P2Y12 inhibitors (*p* = .040, presented in Table 2).

**Table 2:** Procedural and discharge characteristics according to sex and in‐hospital versus out‐of‐hospital STEMI

	In‐hospital STEMI (*n* = 494)			Out‐of‐hospital STEMI (​​​​*n* = 6999)		
	Females (*n* = 158)	Males (*n* = 336)	*p* value	Females (*n* = 1394)	Males (*n* = 5605)	*p* value
Radial access, *n* (%)	52 (32.9)	133 (39.6)	.15	678 (48.6)	3172 (56.6)	<.001
Glycoprotein IIb/IIIa inhibitor, *n* (%)	59 (37.3)	107 (31.8)	.23	447 (32.1)	2208 (39.4)	<.001
Mechanical ventricular support, *n* (%)	13 (8.2)	31 (9.2)	.72	19 (1.4)	201 (3.6)	<.001
Culprit vessel, *n* (%)						
RCA	69 (43.7)	133 (39.6)		659 (47.3)	2192 (39.1)	
LAD	64 (40.5)	142 (42.3)		514 (36.9)	2400 (42.8)	
LCx	19 (12.0)	42 (12.5)	.41	196 (14.1)	912 (16.3)	<.001
Left main	4 (2.5)	5 (1.5)		17 (1.2)	72 (1.3)	
Graft	2 (1.3)	14 (4.2)		8 (0.6)	29 (0.5)	
Complex lesion, *n* (%)	119 (75.3)	247 (73.5)	.67	899 (64.5)	3729 (66.5)	.15
Stent thrombosis, *n* (%)	27 (17.1)	55 (16.4)	.84	33 (2.4)	184 (3.3)	.078
Number of stents implanted	1.09 ± 0.65	1.10 ± 0.66	.92	1.15 ± 0.52	1.18 ± 0.53	.04
Drug‐eluting stent, *n* (%)	110 (69.6)	234 (69.6)	1.00	1045 (75.0)	4291 (76.6)	.21
Procedural success, *n* (%)	151 (95.6)	310 (92.3)	.17	1307 (93.7)	5303 (94.6)	.21
Length of stay (days)	12.6 (±31.3)	8.3 (±8.4)	.019	5.1 (±4.6)	4.8 (±4.7)	.028
Referral to cardiac rehabilitation, *n* (%)	99 (71.2)	228 (76.2)	.06	1047 (81.5)	4491 (85.1)	.002
Discharge medication, *n* (%)						
Aspirin	133 (96.4)	282 (94.6)	.43	1248 (97.7)	5161 (98.2)	.30
Clopidogrel	60 (43.5)	93 (31.2)	.013	378 (29.6)	1424 (27.1)	.070
Ticagrelor	75 (54.3)	192 (64.6)	.040	864 (67.8)	3742 (71.2)	.016
Beta blockers	109 (79.6)	229 (77.4)	.61	1096 (86.0)	4639 (88.4)	.021
ACE/ARB	93 (67.9)	214 (72.1)	.37	1049 (82.3)	4477 (85.3)	.008
Statin	121 (88.3)	280 (94.3)	.030	1215 (95.4)	5123 (97.6)	<.001

Abbreviation: ACE/ARB, angiotensin converting enzyme inhibitors/angiotensin‐receptor blockers;LAD, left anterior descending; LCx, left circumflex; RCA, right coronary artery; STEMI, ST‐elevation myocardial infarction.

**Figure 2:**
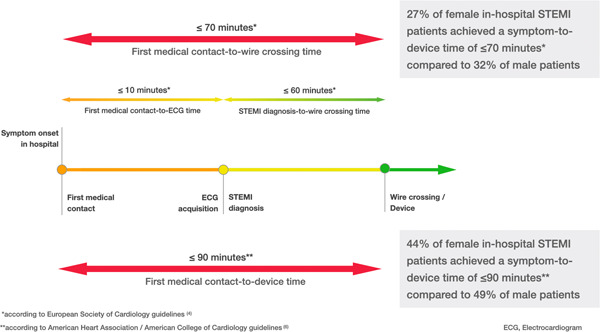
Proportion of female and male in‐hospital STEMI patients achieving guideline‐recommended revascularization times. Female patients had significantly longer unadjusted (119.4 vs. 105.2 min, *p* < .001) and adjusted (104.6 vs. 94.3 min, *p* < .001) geometric mean STD time than male patients. Only 27% of female in‐hospital STEMI patients achieved an STD time of ≤70 min compared with 32% of male patients. Only 44% of female in‐hospital STEMI patients achieved an STD time ≤90 min compared with 49% of male in‐hospital STEMI patients. STD, symptom‐to‐device; STEMI, ST‐elevation myocardial infarction

Female patients had significantly longer unadjusted (119.4 vs. 105.2 min, *p* < .001) and adjusted (104.6 vs. 94.3 min, *p* < .001) geometric mean STD time than male patients. Only 27% of female in‐hospital STEMI patients achieved an STD time of ≤70 min compared with 32% of male patients (displayed in Figure 2).^4^ Only 44% of female in‐hospital STEMI patients achieved an STD time ≤90 min compared with 49% of male in‐hospital STEMI patients.^6^

Female patients presenting with in‐hospital STEMI had numerically worse outcomes at 12‐months (all‐cause mortality: 27.1% vs. 20.3%, *p* = .92) and 30 days (all‐cause mortality: 13.9% vs. 11.9%, *p* = .43; MACE: 22.8% vs. 19.3%, *p* = .87; MACCE: 24.1% vs. 20.8%, *p* = 1; major bleeding: 5.7% vs. 4.5%, *p* = .79; new heart failure: 3.8% vs. 1.8%, *p* = .14; recurrent MI: 8.6% vs. 5.7%, *p* = .43) compared with male patients (shown in Table S1). However, the differences were not statistically significant. In the multivariable models, including the interaction analysis for sex impact, there was no relationship between sex and in‐hospital symptom onset for 12‐month mortality (shown in Table S2) and 30‐day mortality, MACE, MACCE, and major bleeding (not shown in table). Female sex was not associated with poorer 12‐month mortality on multivariable analysis (OR: 1.26, 95% CI: 0.94 – 1.67, *p* = .12).

## 4 DISCUSSION

This is the first study to assess sex differences in patients with an in‐hospital STEMI, utilizing a large registry that captured all STEMI patients who were treated with PCI. The main findings of our study were that: (i) women comprised a larger proportion of patients with in‐hospital STEMI compared with out‐of‐hospital STEMI; (ii) women with in‐hospital STEMI had significantly longer adjusted ischemic times compared with men and received significantly less Ticagrelor and statins; (iii) there was no significant interaction between sex and in‐hospital symptom onset for 12‐month and 30‐day mortality, MACE, MACCE, and major bleeding and (iv) female sex was not independently associated with higher 12‐month mortality for in‐hospital STEMI patients.

Two groups of patients with STEMI are known to be distinct in their characteristics: women and patients with in‐hospital STEMI. Both groups have been described to suffer worse outcomes after a STEMI.^12^, ^15^, ^18^ However, data on in‐hospital STEMI are scant and no previous studies have addressed sex differences in this unique cohort. This is surprising given that patients who are hospitalized for a noncardiac cause have a 40−50 times higher likelihood of suffering a STEMI compared with people in the community.^30^, ^31^ In our cohort, 6.6% of all STEMI cases occurred in patients already admitted to the hospital, consistent with the 5%−8% described in previous literature.^15^, ^22^, ^32^ Of interest, women made up a larger proportion (31.9% vs. 19.9%) of patients with in‐hospital STEMI, compared with standard out‐of‐hospital STEMI. As a result, identifying sex differences is important.

Interestingly, sex discrepancies in baseline characteristics were not as evident in patients with in‐hospital STEMI compared with out‐of‐hospital STEMI patients. Equally so, peri‐procedural characteristics were not significantly different between men and women with in‐hospital STEMI, which is unlike the sex difference observed in patients with out‐of‐hospital STEMIs.

Female in‐hospital STEMI patients had higher absolute rates of MACE, MACCE, all‐cause mortality, and major bleeding; however, they did not reach statistical significance, likely due to the small sample size.

The significant sex difference identified for female patients with in‐hospital STEMI was a mean 10‐min delay from symptom onset to reperfusion when compared with males. Importantly, this difference was evident even after adjustment for confounders. Significantly longer ischemic times in females compared with males in the general STEMI cohort are well described, largely driven by delays in symptom onset to FMC or hospital arrival.^16^, ^17^, ^18^, ^33^, ^34^ In our inpatient STEMI cohort, significant sex delays were seen, despite patients being already admitted to the hospital. However, similar factors may be at play. Females experience a higher rate of associated symptoms and therefore healthcare providers are less likely to attribute their chest pain to a STEMI compared with males.^18^, ^34^, ^35^ The potential for both patient and professional bias is also possible, with a lower perception of females' risk for MI.^16^ The ESC guidelines state that patients with suspected STEMI should receive an ECG within 10 min of FMC with an ECG‐to‐device time ≤60 min.^4^ This would equate to an STD time of ≤70 min for patients with in‐hospital STEMI.^4^ If we were to use the same criteria for our female patients with in‐hospital STEMI, only 27% achieved the recommended STD time of ≤70 min, compared with 32% of males. According to the ACC/AHA,^6^ an STD time ≤90 min was achieved in 44% of female in‐hospital STEMI patients, compared with 49% in male in‐hospital STEMI patients. Similar to out‐of‐hospital patients, check‐lists and protocols ^36^ as well as healthcare professional education, could help establish equal care for females, as for male patients.^20^, ^23^ The current study included only patients with in‐hospital STEMI who were deemed suitable for and underwent PCI. Previous studies that included all‐comers, not just those treated with PCI, demonstrated women made up an even higher proportion of patients with in‐hospital STEMI at 44%−47%.^15^, ^32^ We know that female patients presenting with standard out‐of‐hospital STEMI are less likely to undergo PCI.^17^ Given the 12%−15% difference in female proportions between our study, and those on all‐comers, we speculate that less women with in‐hospital STEMI are being taken to the catheterization laboratory. As to if this is a true sex difference, or a marker of female in‐hospital STEMIs being older with higher comorbid burden, requires further study.

Consistent with studies in all‐comer patients with STEMI, women with an in‐hospital STEMI were less likely to be discharged on Ticagrelor compared with men.^37^, ^38^, ^39^ This is likely due to the higher bleeding risk in women compared with men.^40^, ^41^ However, while studies show that P2Y12 inhibitors lead to more bleeding in women,^42^ this is mostly true for patients with stable CAD.^41^ On the contrary, women with STEMI benefit equally from a potent P2Y12 inhibitor compared with men.^40^, ^41^ In addition, 12% of female patients with an in‐hospital STEMI did not receive high‐dose statin therapy, which was significantly different from males. This is particularly unfortunate given the unequivocally demonstrated benefits of statin therapy in secondary prevention.^43^ While this may reflect underlying comorbid conditions two factors may also explain it: first, patients with in‐hospital STEMI are usually not under the primary care of a cardiologist and, second, treating doctors underestimate women's risk for recurrent events.^44^

## 5 LIMITATIONS

Our study has certain limitations. First, the registry does not provide data about the condition for which patients with in‐hospital STEMI were hospitalized and therefore cannot assess the impact of this on survival. Second, our registry only captures patients with STEMI treated with PCI; hence, we cannot comment on sex differences that occur in admitted patients who did not undergo revascularization, which can lead to selection and survivor bias. However, it is well known that females with STEMI are less likely to undergo PCI,^45^ which would only strengthen our results, since STEMI patients not taken to the catheterization laboratory are likely to have an even poorer outcome. Third, the ability to detect small sex differences was limited by the in‐hospital STEMI sample size.

Lastly, the data were not assessed for multiple recruitments; however, only 5% of stent thromboses resulting in an in‐hospital STEMI occurred during the same admission as an initial PCI.

## 6 CONCLUSIONS

Female In‐hospital STEMI patients suffer from high MACE, mortality, bleeding, and recurrent MI with no statistical difference in outcomes compared with males. However, female patients with in‐hospital STEMI experienced an average 10‐min delay from symptom onset to reperfusion compared with males, after adjustment for confounders. Raising awareness of sex differences in this under‐studied population of patients with in‐hospital STEMI is required to improve outcomes and further narrow the gap.

## CONFLICT OF INTERESTS

Julia Stehli is supported by a Monash University scholarship. Ron Dick receives teaching and travel grants from Abbott, Medtronic, and Boehringer. He serves on the advisory board of Astra Zeneca. Stephen J. Duffy is proctor for Medtronic and his work is supported by a grant (1111170) from the National Health and Medical Research Council of Australia. Sarah Zaman is supported by a fellowship (101993) from the National Heart Foundation of Australia. Sarah Zaman has obtained research funding from Abbott Vascular, Biotronik Australia, and Medtronic Australia and speaking honoraria from AstraZeneca, Boehringer Ingelheim, and Amgen and consulting fees from Medtronic. All other authors have no conflict of interests.

## Supporting information

Supporting information.

## Data Availability

The deidentified data analyzed for the purpose of this study are available upon request to the VCOR Data Access, Research and Publications Committee by emailing vcor@monash.edu.
