# Supplementary material for: Sex differences in treatment and outcomes of patients with in‐hospital ST‐elevation myocardial infarction
Source: Clin Cardiol. 2022 Mar 7;45(4):427–34. doi: 10.1002/clc.23797 (PMC9019891; doi:10.1002/clc.23797)
Supplement: Supplementary file 1 — Supporting information. [file CLC-45-427-s001.docx]

***Supplementary Table 1 - Outcomes according to sex for the in-hospital and out-of-hospital cohort***

|  | In-hospital STEMI | | Adjusted* Odds Ratio | 95% CI | p-value | Out-of-hospital STEMI | | Adjusted* Odds Ratio | 95% CI | p-value |
| --- | --- | --- | --- | --- | --- | --- | --- | --- | --- | --- |
|  | Females  n = 158 | Males  n = 336 |  |  |  | Females  n = 1394 | Males  n = 5605 |  |  |  |
| 30-day outcomes |  |  |  |  |  |  |  |  |  |  |
| All-cause mortality, n (%) | 22 (13.9%) | 40 (11.9%) | 0.74 | 0.35 - 1.57 | ***0.43*** | 131 (9.4%) | 365 (6.5%) | 1.32 | 1.02 - 1.71 | ***0.04*** |
| MACE, n (%) | 36 (22.8%) | 65 (19.3%) | 1.05 | 0.61 - 1.81 | ***0.87*** | 170 (12.2%) | 514 (9.2%) | 1.24 | 1.01 - 1.54 | ***0.04*** |
| MACCE, n (%) | 38 (24.1%) | 70 (20.8%) | 1 | 0.59 - 1.70 | ***1*** | 175 (12.6%) | 541 (9.7%) | 1.21 | 0.98 - 1.49 | ***0.08*** |
| Major bleeding, n (%) | 9 (5.7%) | 15 (4.5%) | 1.13 | 0.46 - 2.73 | ***0.79*** | 51 (3.7%) | 113 (2.0%) | 1.65 | 1.16 - 2.34 | ***0.005*** |
| New heart failure, n (%) | 6 (3.8%) | 6 (1.8%) | 2.42 | 0.75 - 7.83 | ***0.14*** | 29 (2.1%) | 71 (1.3%) | 1.38 | 0.88 - 2.16 | ***0.17*** |
| Recurrent MI, n (%) | 12 (8.6%) | 16 (5.7%) | 1.4 | 0.61 - 3.23 | ***0.43*** | 27 (2.2%) | 77 (1.5%) | 1.44 | 0.91 - 2.27 | ***0.12*** |
| New renal impairment, n (%) | 17 (11.6%) | 36 (11.7%) | 0.83 | 0.43 - 1.61 | ***0.58*** | 98 (7.3%) | 310 (5.7%) | 1.01 | 0.79 - 1.31 | ***0.91*** |
| 12-months all-cause mortality, n (%) | 35 (27.1%) | 54 (20.3%) | 0.97 | 0.51 - 1.82 | ***0.92*** | 182 (16.3%) | 447 (10.0%) | 1.56 | 1.24 - 1.96 | ***<0.001*** |

***Supplementary Table 2 – Multivariable Predictors of 12-month mortality in overall STEMI population***

|  | Odds Ratio | 95% CI | p-value |
| --- | --- | --- | --- |
| Interaction of female sex with in-hospital symptom onset | 0.73 | 0.35 - 1.54 | ***0.41*** |
| Female sex | 1.26 | 0.94 – 1.70 | ***0.13*** |
| In-hospital symptom onset | 2.00 | 1.25 – 3.20 | ***0.004*** |
| Age, each year increase | 1.03 | 1.02 – 1.04 | ***<0.001*** |
| Diabetes | 1.08 | 0.79 – 1.47 | ***0.67*** |
| Previous PCI or CABG | 1.08 | 0.75 – 1.56 | ***0.65*** |
| Cerebrovascular disease | 2.00 | 1.36 – 2.92 | ***<0.001*** |
| Cardiogenic shock and/or cardiac arrest | 10.28 | 7.9 – 13.3 | ***<0.001*** |
| Left ventricular ejection fraction <35% | 7.53 | 5.34 – 10.6 | ***<0.001*** |
| Estimated glomerular filtration rate | 0.98 | 0.98 – 0.99 | ***<0.001*** |
| Graft lesion | 1.48 | 0.58 – 3.74 | ***0.41*** |
| Stent thrombosis | 1.02 | 0.56 – 1.87 | ***0.94*** |
